# Crystal structure and structure-based mutagenesis of actin-specific ADP-ribosylating toxin CPILE-a as novel enterotoxin

**DOI:** 10.1371/journal.pone.0171278

**Published:** 2017-02-15

**Authors:** Waraphan Toniti, Toru Yoshida, Toshiharu Tsurumura, Daisuke Irikura, Chie Monma, Yoichi Kamata, Hideaki Tsuge

**Affiliations:** 1 Department of Bioresource and Environmental Sciences, Faculty of Life Sciences, Kyoto Sangyo University, Kyoto, Japan; 2 Institute for Protein Dynamics, Kyoto Sangyo University, Kyoto, Japan; 3 Advanced R&D Center, Horiba, Ltd., Kyoto, Japan; 4 Department of Microbiology, Tokyo Metropolitan Institute of Public Health, Tokyo, Japan; 5 Department of Veterinary Medicine, Iwate University, Morioka, Japan; Institut Pasteur, FRANCE

## Abstract

Unusual outbreaks of food poisoning in Japan were reported in which *Clostridium perfringens* was strongly suspected to be the cause based on epidemiological information and fingerprinting of isolates. The isolated strains lack the typical *C*. *perfringens* enterotoxin (CPE) but secrete a new enterotoxin consisting of two components: *C*. *perfringens* iota-like enterotoxin-a (CPILE-a), which acts as an enzymatic ADP-ribosyltransferase, and CPILE-b, a membrane binding component. Here we present the crystal structures of apo-CPILE-a, NAD^+^-CPILE-a and NADH-CPILE-a. Though CPILE-a structure has high similarity with known iota toxin-a (Ia) with NAD^+^, it possesses two extra-long protruding loops from G262-S269 and E402-K408 that are distinct from Ia. Based on the Ia–actin complex structure, we focused on actin-binding interface regions (I-V) including two protruding loops (PT) and examined how mutations in these regions affect the ADP-ribosylation activity of CPILE-a. Though some site-directed mutagenesis studies have already been conducted on the actin binding site of Ia, in the present study, mutagenesis studies were conducted against both α- and β/γ-actin in CPILE-a and Ia. Interestingly, CPILE-a ADP-ribosylates both α- and β/γ-actin, but its sensitivity towards β/γ-actin is 36% compared with α-actin. Our results contrast to that only C2-I ADP-ribosylates β/γ-actin. We also showed that PT-I and two convex-concave interactions in CPILE-a are important for actin binding. The current study is the first detailed analysis of site-directed mutagenesis in the actin binding region of Ia and CPILE-a against both α- and β/γ-actin.

## Introduction

Human food-borne gastroenteritis caused by the *Clostridium perfringens* enterotoxin (CPE) has been widely studied. *C*. *perfringens* is a Gram-positive, rod-shaped, anaerobic, spore-forming, pathogenic bacterium of the genus Clostridium that can be classified into five types (A–E) based on the type of toxin secreted. The CPE is known to cause diarrhea in humans and other animals. Most CPE-producing *C*. *perfringens* are classified as type A. On the other hand, type E produces alpha and iota toxins and leads to antibiotic–associated enterotoxemia in bovine and ovine species [1].

Type E *C*. *perfringens* specifically produces an iota toxin consisting of two unlinked components, iota-a (Ia) and iota-b (Ib). Ia ADP-ribosylates globular actin at Arg177, leading to destruction of filamentous actin and the intoxicated cell. Ib binds Ia and host cell membrane receptors which facilitate translocation of the two components into the cell. Iota-like binary toxins are produced by *C*. *botulinum* (C2), *C*. *difficile* (CDT), and *C*. *spiroforme* (CST). These toxins are structurally related to vegetative insecticidal protein (VIP) produced by *Bacillus cereus* and *B*. *thuringiensis*, which consists of two similar N-terminal and C-terminal domains [2, 3]. The N-terminal domain and the C-terminal domain bears binding with the membrane binding component and ADP-ribosyltransferase, respectively [4, 5].

Unusual outbreaks of food poisoning in Japan were reported in which non-CPE producing *C*. *perfringens* strain was strongly suspected to be the cause based on epidemiological information and fingerprinting of isolates [6–8]. The *C*. *perfringens* W5052 strain isolated from the food poisoning outbreak in Tokyo was shown to induce the death of both Vero and enterotoxin receptor-lacking L929 cells. This strain also caused swelling and fluid accumulation of the ileal loop of rabbits. This newly isolated strain lacks the *cpe* gene and did not produce any known enterotoxin. Instead, a novel enterotoxin was reported as a CPILE (*C*. *perfringens* iota-like enterotoxin) [7, 9]. While binary *C*. *perfringens* enterotoxin (BEC) was found in strain OS1 (Osaka) and TS1 (Tochigi) outbreaks [8], it was shown the two toxins CPILE and BEC were identical based on the results of a BLAST search [7].

The novel enterotoxin of *C*. *perfringens* W5052 consists of two unlinked components: CPILE-a, which acts as an enzymatic ADP-ribosyltransferase (ART), and CPILE-b, a membrane binding and pore forming component [7]. CPILE-a is composed of 419 amino acid residues with a molecular mass of 47.5 kDa, whereas CPILE-b is composed of 799 amino acids residues with a molecular mass of 91.1kDa (DDBJ/EMBL/FASTA accession numbers AB921559 and AB921560). While neither CPILE-a nor untrypsinized CPILE-b are able to round or kill Vero or L929 cells, trypsin-treated CPLIE-b can. These findings demonstrate prominent differences between CPILE-b and Ib from the known iota toxin. Ib shows cytotoxicity in A431 and A549 cells and causes a rapid necrosis, but Vero cells were not susceptible to Ib [10, 11], suggesting the presence of distinct receptors for the members of the iota toxin group. Lipolysis-stimulated lipoprotein receptor was identified as the receptor for Ib [12, 13]. Moreover, a mixture of CPILE-a and CPILE-b has been shown to enhance cell rounding and killing activity [7, 8]. Hence, these results suggest CPILE is a unique and novel enterotoxin produced by *C*. *perfringens*.

ADP-ribosylation is a well-known posttranslational modification in which the ADP-ribose moiety is transferred from NAD^+^ to a targeted amino acid on the target protein after nicotinamide is released. This reaction is catalyzed by a superfamily of ARTs found in prokaryotes and eukaryotes, including humans. Based on their target specificity, bacterial ARTs have been traditionally classified into four types: type I includes cholera toxin, which ADP-ribosylates Gsα [14], and pertussis toxin, which ADP-ribosylates both Gi and Go [15–17]; type II includes diphtheria toxin [18, 19] and exotoxin A from *Pseudomonas aeruginosa* [20, 21], which modify elongation factor 2; type III includes the C3 toxin from *C*. *botulinum*, which ADP-ribosylates RhoA [22]; type IV includes iota toxin from *C*. *perfringens*, which ADP-ribosylates actin [23]. All of these toxins possess three important motifs R/H, S-T-S, and E/Q-X-E. The last motif is on the ADP-ribosylating turn–turn loop (ARTT-loop: φ-X-X-(E/Q)-X-E) which is the crucial substrate recognition loop proposed by Han et al [24–28]. Type IV ARTs have been identified as an enzymatic unit of binary toxins, including Ia, CDT component a (CDTa), and C2-I [29]. Interestingly, type IV and III toxins are structurally similar, but their targets and modified residues are totally different; type IV toxins (e.g., Ia) ADP-ribosylate Arg177 of actin, while type III toxins (e.g., C3) ADP-ribosylate Asn41 of RhoA [30].

Recent structural and functional analyses of Ia (type IV) -actin complex have revealed [31, 32] that Ia's fives loops [loop I (Tyr60-Tyr62), loop II (R248-Y251: active site loop), loop III (G298-Y311), loop IV (S347-R352: phosphate-nicotinamide (PN) loop), loop V (G374-E380:ADP-ribosylating turn turn (ARTT) loop] are important for the recognition of α-actin [31, 32]. Interestingly, loop I on the N-terminal domain is important for the actin binding. Though α-actin is recognized by Ia without any large conformational change [31, 32], the structural and functional analysis of C3 (type III)-RhoA revealed that both C3 and RhoA need to adopt a conformational change in order to recognize each other [33]. The ARTT-loop is very important for recognition of substrate proteins and modified residues; Tyr180 (φ: an aromatic residue on the first turn of the ARTT-loop) of the former turn binds RhoA via hydrophobic interaction, and Gln183 of the latter turn is crucial for recognition of modified residue Asn41 of RhoA. The complex structure of C3-RhoA shows the ARTT-loop is crucial for substrate recognition. However, in the Ia–actin complex, ARTT-loop recognition has not yet been confirmed. Furthermore, there is a much greater variety of amino acids that are ADP-ribosylated by other ARTs, such as pertussis toxin modifies Cys residue and novel *P*. *luminescens* ART TccC3 modifies Thr residue [34]. Thus, which factors determine the specificity of the ARTs requires further clarification [35].

The current study of CPILE-a sought to reveal not only NAD^+^ binding site by crystallographic study but also actin binding site by structural-based mutagenesis study. We revealed three structures of apo-CPILE-a, NAD^+^-CPILE-a and NADH-CPILE-a. We conducted detailed analysis of actin ADP-ribosylation by each CPILE-a and Ia against both α- and β/γ-actin. These characterizations show that newly found CPILE-a is a unique ART which has different structural and substrate recognition features from Ia. However, it suggests that the main strategy of substrate recognition using two convex-concave interactions is common in all the family toxins.

## Materials and methods

### Protein expression and purification

The CPILE-a/pGEX4T-2 vectors which expressed a glutathione-*S*-transferase with an N-terminal thrombin site were transformed into *Escherichia coli* BL21 (DE3)-competent cells. A single colony was selected, incubated in LB broth containing 100 μg/mL ampicillin, and grown overnight at 37°C with shaking at 150 rpm. Then, the overnight culture was inoculated into 1.5 L of LB broth containing ampicillin. The culture was allowed to grow until the absorbance at 600 nm reached 0.6, and then 1 mM isopropyl β-D-1-thiogalactopyranoside was added. The cultures were incubated at 16°C with 100 rpm shaking overnight and were harvested the next day by centrifugation at 8000 × *g* for 5 min at 4°C. CPILE-a protein was extracted using Bugbuster^00AE^ protein extraction reagent (Novagen, WI, USA), followed by centrifugation at 13,000 rpm for 35 min at 4°C. After, the supernatant was added to Glutathione Sepharose 4B beads (GE Healthcare, UK). The beads were washed with phosphate-buffered saline prior to adding thrombin, then the suspension was incubated at room temperature overnight. CPILE-a protein was eluted with phosphate-buffered saline and further purified by gel filtration chromatography using a Superdex 200 column (GE Healthcare, UK). The purity of CPILE-a was verified by sodium dodecyl sulfate-polyacrylamide gel electrophoresis (SDS-PAGE). Twelve mutants of CPILE-a were expressed and purified as same as the wildtype did. Expression and purification of Ia and its mutants were described before [5].

### Crystallization and structure determination

CPILE-a crystals were grown at 4°C using a hanging drop vapor diffusion method against a reservoir containing 18% PEG4000, 100 mM Tris HCl (pH 8.5), and 200 mM MgCl_2_. The hanging drop vapor diffusion method was performed with equal volumes of 13 mg/ml CPILE-a and reservoir solution. Thirteen mg/ml CPILE-a was co-crystallized with either 10 mM NAD^+^- or NADH and the complex crystals were grown at 4°C using a hanging drop vapor diffusion method against a reservoir containing 18% PEG4000, 100 mM Tris HCl (pH 8.5), and 200 mM MgCl_2_ as same as the apo-CPILE-a.

The selected crystals were flash-frozen in liquid nitrogen with 30% ethylene glycol as a cryoprotectant, and data were collected in-house using a MicroMax-007 generator and R-AXIS VII X-ray diffractometer (Rigaku) at 2.01 Å. All diffraction images were processed by HKL2000 suite [36]. However, the first attempt to reveal the structure of CPILE-a by molecular replacement failed even though Ia’s structure (1GIQ) was used as a search model. Thus, three Cys substitutions (A97C, S185C, and S366C) were introduced into CPILE-a for heavy atom soaking. The crystals were soaked in mother liquor with 1 mM HgCl_2_ for 22 h, then data sets at three different wavelengths for peak, edge, and remote were collected using a NW-12A beamline in a KEK Photon Factory-Advanced Ring. The initial phase was obtained with SHELX (hkl2map) using single anomalous dispersion [37]. Autotracing was done with ARP/wARP following iterative manual–tracings [38]. The structure was refined using phenix.refine in the Phenix.refine suite [39], Coot [40], and PDB_REDO Web server [41]. The structure was further refined against wild type CPILE-a data obtained earlier in-house at 2.01 Å resolution and an *I*222 space group with the following cell dimensions: a = 70.3 Å, b = 100.7 Å, and c = 126.0 Å. The structures of NAD^+^-CPILE-a and NADH-CPILE-a were solved by molecular replacement using apo-CPILE-a structure at 1.80 Å and 2.26 Å, respectively.

### Mutagenesis and ART activity assay

CPILE-a residues involved in actin binding were selected according to the actin-CPILE-a model. Eleven single amino acids were selected for substitution by Ala, including L61A and N63A (loop I), Y252A (loop II), L308A and Y313A (loop III), K353A (loop IV), Y377A, E380A and E382A (loop V), and S404A L405A (PT-II). Moreover, a double Gly substitution was made at E266GN267G on PT-I. After purification, these mutants were tested for ART activity against both α- and β/γ-actin using FITC-labeling as a part of the streptavidin-biotin complex.

CPILE-a, α-actin, β/γ-actin, and biotin-NAD^+^ were diluted with G-buffer (General G-actin buffer: 2 mM Tris-HCl pH 8.0, 0.2 mM CaCl_2_, 0.2 mM ATP, and 0.5 mM dithiothreitol). Assay solution contained 0.3 μM CPILE-a, 3.0 μM actin, and 3.75 μM biotin-NAD^+^. After the assay solution was incubated 37°C for 10 min, the sample buffer for SDS-PAGE was added and they were heated 98°C for 3 min. These samples were subjected to SDS-PAGE. After the gel was fixed in fixing solution and washed three times with PBS, the gel was stained with streptavidin-FITC solution (1:1000) for 16 h. The gel was washed three times with PBS, and the FITC labeled actin was detected by Typhoon FLA9000 (GE Healthcare) at 500 V. In α-actin case, two bands were observed [α-actin (top) and protease cleaved α-actin (bottom)]. Thus total intensities of FITC- labeled actins from both bands were calculated.

ART assays of Ia mutants were also completed. Some mutants were confirmed again (Y60S, Y62S, R251A, E378S, and E380A) [5, 31, 32], and L306S, Y311S, K351S and Y375S mutants were newly determined in this study. All these mutants were selected based on Ia-actin structure because they are located on the binding surface with actin. This time, however, all of the assays were completed against both α- and β/γ-actin. α-Actin (rabbit skeletal muscle) and β/γ-actin (human platelet) were purchased from Cytoskeleton (Denver, CO, USA). Streptavidin-FITC was purchased from Vector Laboratories.

### NADase activity assay

Assay solution contained 10 μM CPILE-a or Ia, 10 μM actin, 1mM NAD^+^ and 20mM Tris-HCl (pH 8.0). NADase activity was measured at 260 nm using a TSKgel ODS-80Tm column (Tosoh). Reactions were carried out at 37°C 10 min, at 25°C for 1 h and overnight. The eluent was 20 mM phosphate buffer (pH 5.9) with 5% acetonitrile.

### Molecular modeling of CPILE-a-actin complex

CPILE-a-α-actin complex were modeled based on the Ia−actin complex (4H03). Complex model was obtained using LSQ superpose in CCP4i suite [42]. Finally, energy minimization, which consists of steepest descent and conjugate gradient methods, was applied for finding a minimum on the potential energy of the complex by UCSF chimera [43].

## Results

### Crystal structure of CPILE-a

**Fig 1:**
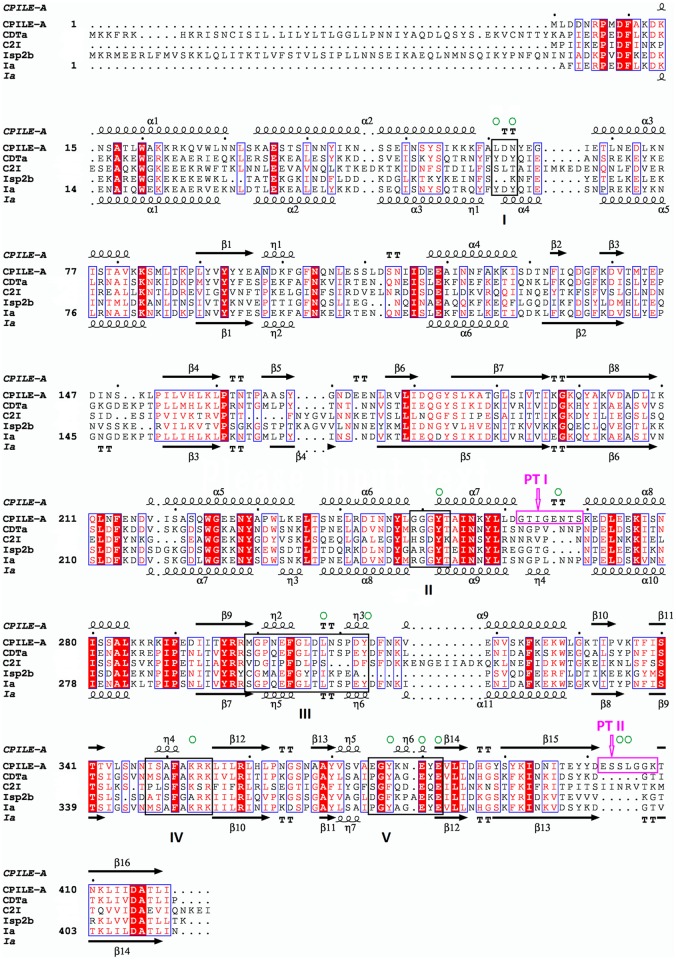
Sequence alignment of CPILE-a, CDTa, C2-I, Isp2b, and Ia. Black square boxes represent the five interaction loop (I–V). Hot pink boxes designate PT-I and PT-II. Green circles show selected amino acid residues for mutational studies. Secondary structures are shown for CPILE-a (top) and Ia (bottom).

**Fig 2:**
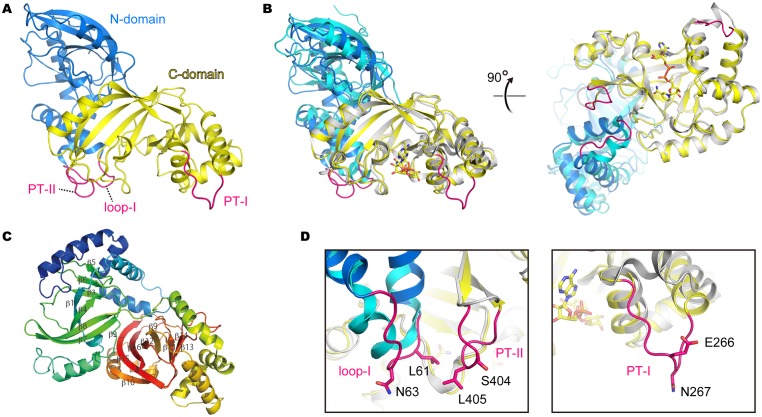
Crystal structure of CPILE-a. **A,** Overall structure of NAD^+^-CPILE-a. The N-terminal (residues 3–211) and the C-terminal (residues 212–419) domains are shown in blue and yellow, respectively. PT-I, PT-II, and loop I are shown in magenta. **B,** Superimposed structures of CPILE-a and Ia (4H03). The N- and C-terminal domains are shown in cyan and white, respectively. NAD^+^ is shown as a stick. C, Color is a rainbow ramp from purple at the N-terminal domain to red from C-terminal domain. β-sheets are labeled. **D,** Close-up views of PT-II and loop I (left) and PT-I (right). Amino acid residues selected for mutational studies (Fig 5) are shown as sticks.

Among the type IV ARTs of interest, the amino acid sequence of CPILE-a showed higher identity with Ia (45%) and CDTa (44%) than with C2-I (28%) and Isp2b (34%) (Fig 1). Thus, the Ia-NADH structure (1GIQ) was used as a template model of molecular replacement (MR), however, the analysis failed. As there are at least two crystals belong to different space groups [*I*222 (*I*212121) and *C*222 (*C*2221)] in the same crystallization conditions, we could not find whether the cause is either poor model or space group. Thus, we tried to determine the phase by single anomalous dispersion (SAD) using triple Cys mutants. Using mercury-soaked crystal data, the structure of CPILE-a was solved using SHELX against the single anomalous dispersion data set at 2.28 Å resolution. The fragment main-chain tracing by SHELXE was excellent (FOM = 0.65; pseudo-free CC = 69.6%). Then, further main-chain tracing, including side-chains, using ARP/wARP was done. Using the triple Cys mutant structure as a template, the final structure of wild type CPILE-a was refined at 2.01 Å resolution (Table 1; Fig 2A and 2B).

**Table 1:** Data collection and refinement.

	CPILE-a	3C Mutated CPILE-a	CPILE-a_NAD^+^	CPILE-a_NADH
**Data collection**				
**Space group**	*I* 222	*I* 222	*I* 222	*I* 222
**Cell dimensions**				
**a, b, c (Å)**	70.3, 100.7, 126.0	70.2, 101.5, 126.5	70.0, 95.2, 125.1	70.6, 101.6, 125.3
**α β, γ(°)**	90.0, 90.0, 90.0	90.0, 90.0, 90.0	90.0, 90.0, 90.0	90.0, 90.0, 90.0
**X-ray source**	MicroMax-007, RAXIS VII (RIGAKU)	PF-AR NW 12A	PF-AR NW 12A	MicroMax-007, RAXIS VII (RIGAKU)
**Wavelength (Å)**	1.5418	1.00697	1.00000	1.5418
**Resolution range (Å)**	50.00–2.01 (2.04–2.01)	50.00–2.20 (2.24–2.20)	50.00–1.80 (1.83–1.80)	50.00–2.26 (2.30–2.26)
**Observed reflections**	198493	255478	242720	127629
***R*meas**†	0.099 (0.660)	0.098 (0.491)	0.152 (0.844)	0.084 (0.408)
***R*pim**‡	0.037 (0.262)	0.041 (0.206)	0.060 (0.370)	0.034 (0.169)
**CC_1/2_**	(0.885)	(0.927)	(0.655)	(0.948)
***I* / σ*I***	31.2 (2.9)	29.8 (5.8)	12.3 (0.92)	41.3 (6.5)
**Completeness (%)**	98.3 (85.8)	100.0 (100.0)	99.4 (92.1)	98.4 (95.0)
**Redundancy**	6.7 (5.2)	5.8 (5.7)	6.3 (4.8)	6.0 (5.4)
**Refinement**				
**Resolution (Å)**	21.8–2.01	42.65–2.20	47.61–1.80	23.48–2.25
***R*work*/R*free §^1^ (%)**	17.3 / 22.6	21.5 / 27.5	19.0 / 23.8	17.7 / 24.8
**Overall *B* factors (Å^2^)**	44.0	-	32.0	47.0
**r.m.s.deviations**				
** Bond lengths (Å)**	0.007	-	0.007	0.007
** Bond angles (°)**	1.084	-	1.124	1.166
**Ramachandran plot**				
** Favored regions (%)**	97.35	93.29	96.39	95.20
** Allowed regions (%)**	2.41	5.04	3.13	4.32
** Outliers (%)**	0.24	1.68	0.48	0.48
**PDB ID**	5GTT	-	5WTZ	5WU0

^†$R\text{meas}=\sum_{hkl}{\left\{N\left(hkl\right)/\left[N\left(hkl\right)-1\right]\right\}}^{\frac{1}{2}}\sum_{i}\left|I_{i}\left(hkl\right)-\langle I\left(hkl\right)\rangle \right|/\sum_{hkl}\sum_{i}I_{i}\left(hkl\right)$^,

^‡$R\text{pim}=\sum_{hkl}{\left\{1/\left[N\left(hkl\right)-1\right]\right\}}^{\frac{1}{2}}\sum_{i}\left|I_{i}\left(hkl\right)-\langle I\left(hkl\right)\rangle \right|/\sum_{hkl}\sum_{i}I_{i}\left(hkl\right)$^, where *I*_*i*_(*hkl*) are the observed intensities, 〈*I* (*hkl*)〉 is the average intensity and *N*(*hkl*) is the multiplicity of reflection *khl*.

CC_1/2_ = percentage of correlation between intensities from random *hkl*-data sets

^§1$R\text{work}=\sum_{hkl}\left|\left|F_{obs}\right|-\left|F_{calc}\right|\right|/\sum_{hkl}\left|F_{obs}\right|$^. *R*free is the cross-validation R factor for the test set (5%) of reflections omitted from model refinement.

The CPILE-a crystal belongs to space group *I*222 and consists of one molecule in an asymmetric unit. The structure of CPILE-a can be divided into two domains, N-terminal (amino acids 3–211) and C-terminal (amino acids 212–419). Even though they had a low sequence identity (29%), the structure of N-terminal and C-terminal domain are homologous (1.3 Å RMSD / 133 Cα atoms). Both domains consist of mixed α/β structures and each core domains consist of a β-sandwich core formed by a five-stranded mixed β -sheet (β2- β7- β8- β4- β1 in N-terminal domain and β10- β15- β16- β12- β9 in C-terminal domain) and a three-stranded anti-parallel β -sheet (β3- β8- β5 in N-terminal domain and β11- β14- β13 in C-terminal domain) (Fig 2C). There was a long loop at the junction between the N- and C-terminal domains. The overall structure of CPILE-a was similar to that of other type IV ARTs. These RMSDs were shown in Table 2. Ia has been studied extensively for a long time as the representative ART, especially the complex structure of Ia-actin has been revealed, we compared the structure with Ia. Comparison to Ia, CPILE-a contained a flexible loop (A60-I67). The corresponding region in Ia (Y60-E65) forms an α-helix (loop I). Moreover, two protruding loops in CPILE-a were not seen in Ia: G262-S269 and E402-K408, which are considered to be crucial regions for the binding of actin as described below. Thus, these two loops were named PT-I (G262-S269) and PT-II (E402-K408). These are three distinct structural features between CPILE-a and Ia (Fig 2D).

**Table 2:** Structural comparison to CPILE-a with other ARTs.

	5GTT	5WTZ	5WU0	1GIQ	2J3Z	2WN4	1QS2
5GTT	-						
5WTZ	0.43 (379)	-					
5WU0	0.33 (371)	0.23 (356)	-				
1GIQ	1.56 (360)	1.88 (374)	1.71 (367)	-			
2J3Z	1.92 (336)	1.90 (336)	1.91 (335)	2.52 (328)	-		
2WN4	1.43 (322)	1.71 (337)	1.61 (336)	0.80 (342)	2.07 (280)	-	
1QS2	2.78 (349)	2.82 (343)	2.81 (346)	2.76 (339)	3.73 (348)	1.61 (263)	-

RMSD (Å) and number of the Cα compared in the parenthesis.

5GTT: apo-CPILE-a, 5WTZ: CPILE-a-NAD^+^, 5WU0: CPILE-a-NADH, 1GIQ: Ia, 2J3Z: C2I, 2WN4: CDTa, 1QS2: VIP2

**Fig 3:**
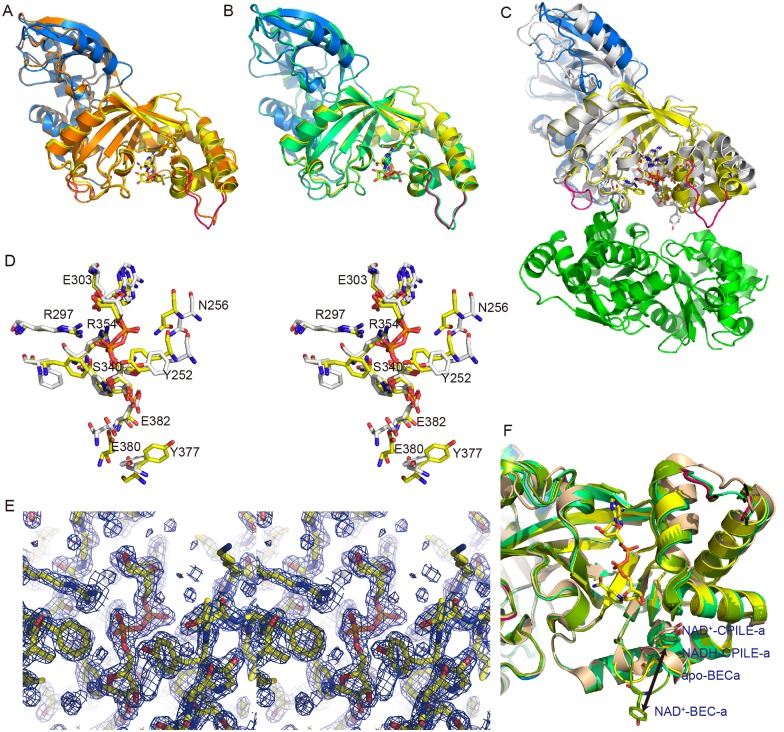
Structure comparison with apo-CPILE-a, NAD^+^-CPILE-a, NADH-CPILE-a and close-up view of NAD-binding site of CPILE-a, Ia and BECa. **A,** Superimposed structures of NAD^+^-CPILE-a and apo-CPILE-a indicated that the binding to NAD^+^ did not induce conformational change to CPILE-a. The N-terminal domain and the C-terminal domain of NAD^+^-CPILE-a are shown in marine and yellow, respectively. The apo-CPILE-a is shown in orange. **B,** Superimposed structures of NAD^+^-CPILE-a and NADH-CPILE-a (lime green) showed very similar structure with r.m.s.d. 0.23 (Å). **C,** Superimposed structures of NAD^+^-CPILE-a and Ia (4H03). Ia and actin showed in white and green, respectively. **D,** Stereo-view of NAD^+^-CPILE-a (yellow) and NAD^+^-Ia (4H03) focused on ligand-binding site (NAD^+^). Ten residues represent the close-contact residues to the NAD^+^. All residues labeled here belong to the NAD^+^-CPILE-a. **E,** Stereo-view of the 2Fo-Fc electron density map of the NAD^+^ contoured at 2σ. **F,** Superimposed structure of NADH-CPILE-a, apo-BEC-a (5H03), and NADH-BECa (5H04) focused on ligand binding site. A distinct residue (Tyr377) on the ARTT-loop is shown in stick model. The NAD^+^-CPILE-a, NADH-CPILE-a, apo-BECa, and NADH-BECa are shown in yellow, green, tint, and split pea, respectively.

We revealed two complex structures of NAD^+^-CPILE-a and NADH-CPILE-a at 1.80 Å and 2.26 Å, respectively. There are no obvious structure differences among three structures, apo-CPILE-a, NAD^+^-CPILE-a, and NADH-CPILE-a including PT-I and PT-II (Fig 3A and 3B and Table 3). Based on the superimposed structure of NAD^+^-Ia-actin (4H03) and NAD^+^-CPILE-a, ten residues around NAD^+^ binding site were structurally conserved and they make NAD conformation specific seen in many ARTs (Fig 3C and 3D). These ten residues were also perfectly conserved in CDTa. On the other hand, there were three residues changes in C2I including [P (E303), S (R354), and F (Y377)]. The RMSD value of the structurally conserved ten residues (Y252, N256, R297, E303, S340, F351, R354, Y377, E380, E382) and NAD^+^ between Ia and CPILE-a are 1.24 Å. Two Arg residues (Arg297 and Arg354) contact with two phosphates. The two important motifs, S-T-S (the first serine is S340 in CPILE-a), in which the second Ser is replaced by Thr in CPILE-a, and E (E380)-X-E (E382) are also conserved among the ARTs (Fig 3D). F351 on a phosphate-nicotinamide loop is conserved among the ARTs (Fig 3D). Recently apo-BECa (5H03) and NADH-BECa (5H04) structures were reported by other group [44]. However, no NAD^+^-bound structure has been reported before, which would be more important because NAD^+^ is a real substrate. The structure of NAD^+^-CPILE-a showed that NAD^+^ electron density is clearly visible (Fig 3E). Next, we compared the BECa structures with CPILE-a. It is interesting that ARTT-loop of NADH-BECa is far those of NADH-CPLE-a as well as NAD^+^-CPILE-a and apo-BECa. The RMSD value of ARTT-loop between NADH-CPILE-a and NADH-BECa is 2.7 Å (Fig 3F and Table 4).

**Table 3:** Structural comparison between CPILE-a and BECa (whole enzyme).

	5GTT	5WTZ	5WU0	5H03	5H04
5GTT	-				
5WTZ	0.43 (379)	-			
5WU0	0.33 (371)	0.23 (356)	-		
5H03	0.49 (351)	0.65 (371)	0.63 (366)	-	
5H04	0.29 (364)	0.28 (348)	0.21 (392)	0.55 (347)	-

RMSD (Å) and number of the Cα compared in the parenthesis.

5GTT: apo-CPILE-a, 5WTZ: CPILE-a-NAD^+^, 5WU0: CPILE-a-NADH, 5H03: apo-BECa, 5H04: BECa-NADH

**Table 4:** Structural comparison between CPILE-a and BECa (focus on the ARTT loop; V373-E380).

	5GTT	5WTZ	5WU0	5H03	5H04
5GTT	-				
5WTZ	0.38 (6)	-			
5WU0	0.58 (7)	0.20 (8)	-		
5H03	0.76 (8)	1.31 (8)	1.35 (8)	-	
5H04	3.02 (8)	2.74 (8)	2.68 (8)	3.28 (8)	-

RMSD (Å) and number of the Cα compared in the parenthesis.

5GTT: apo-CPILE-a, 5WTZ: CPILE-a-NAD^+^, 5WU0: CPILE-a-NADH, 5H03: apo-BECa, 5H04: BECa-NADH

**Fig 4:**
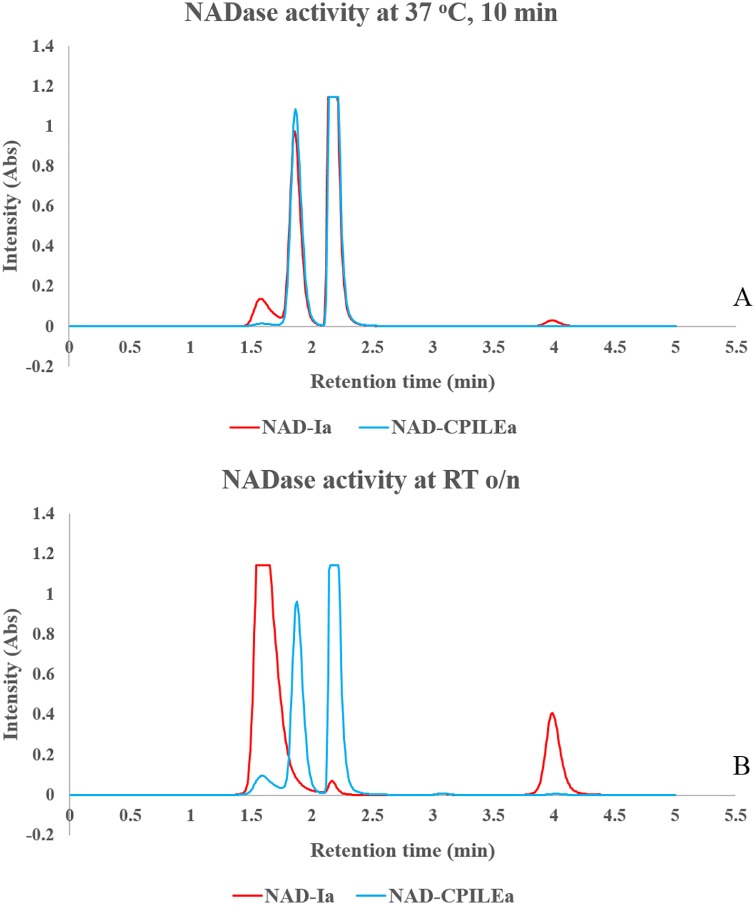
NADase activities of CPILE-a and Ia. **A,** 37°C one hour incubation **B,** 25°C overnight incubation. ADP-ribose and nicotinamide eluted at 1.6 min and 4.0 min, respectively.

Based on these C-terminal domain structural similarity with Ia, we expected CPILE-a to have similar ART and NADase activities. As shown later, CPILE-a shows ART activity against both α- and β/γ-actins. However, there was almost no NADase activity without actin (1 h at 37°C) (Fig 4A), which is completely different from the case for Ia. Even with a longer incubation period (overnight at 25°C), CPILE-a showed low NADase activity (Fig 4B).

### Mutational studies of the CPILE-a actin-binding region

**Fig 5:**
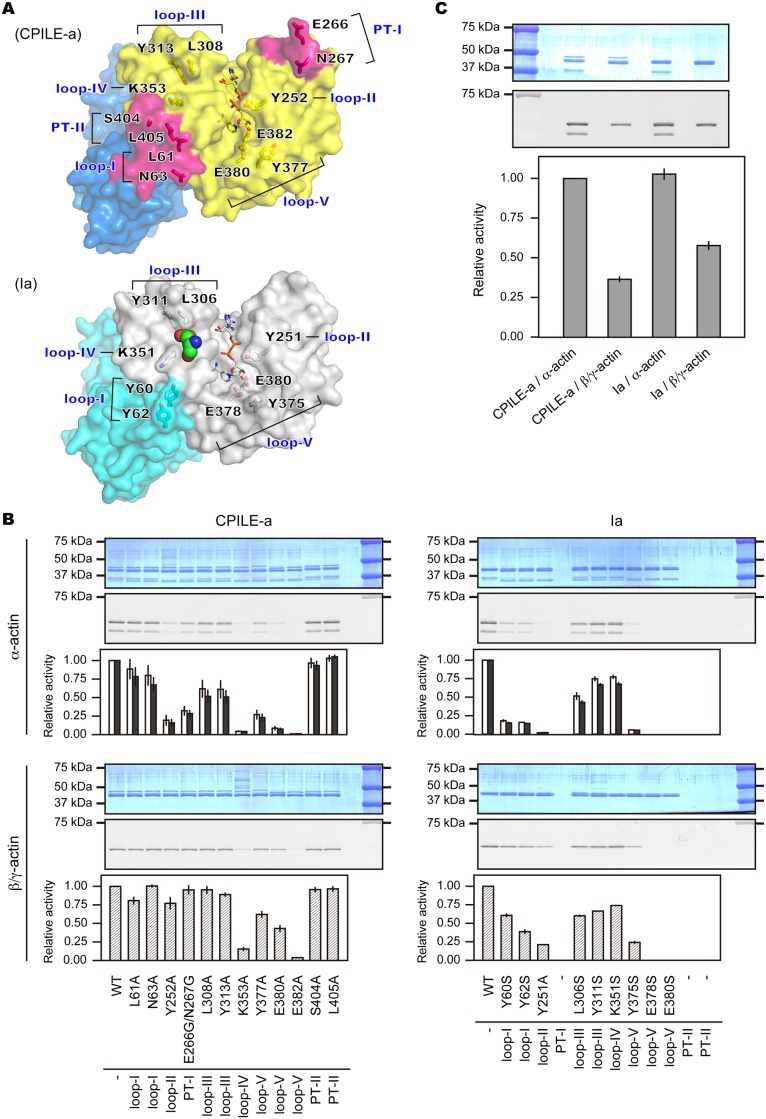
ADP-ribosylation assay of CPILE-a and Ia against α- and β/γ-actin. **A,** the positions of amino acid residues selected for the mutational study. The contact surfaces interacting with actin in CPILE-a (top) and Ia (bottom) are shown. They are color-coded as in Fig 2. The side chain of Glu-270 of actin is shown as sphere in Ia. **B,** comparison of the ADP-ribosylation activity of wildtype (WT) and mutants in CPILE-a (left) and Ia (right). The ADP-ribosylation assay was performed using biotin-NAD^+^ and the biotin-ADP-ribosylated actin was detected by streptavidin-FITC. The SDS-page and the representative scanned images are shown on the top. In α-actin case, two bands were observed [α-actin (top) and protease cleaved α-actin (down)]. Thus each FITC- labeled actin from two bands are calculated and shown (white: non-cleaved black: cleaved). **C,** comparison of the sensitivity of CPILE-a and Ia against α-actin and β/γ-actin. The SDS-PAGE and the representative scanned images are shown on the top. In α-actin case, total FITC-labeled actin from two bands were calculated and shown. The results of three independent experiments were averaged and Error bar represents S.D. in (B) and (C).

Though the structure of the Ia-actin complex has been revealed [31], the substrate-protein recognition mechanism of type IV ARTs is not fully understood. Though we could not obtain a crystal of the CPILE-a-actin complex herein, we assessed the region interacting with actin by comparing it with the structure of the Ia-actin complex, which is the only applicable complex structure for type IV ARTs (Fig 5A). We assumed that five CPILE-a loops were involved in actin binding: loop I (L61-N63 of the N-domain), loop II (G249-T253), loop III (G300-Y313), loop IV (I348-K355), and loop V (E375-E382). Additionally, PT-I and PT-II were assumed to be involved in the actin interface. Based on these assumed interaction regions, CPILE-a mutants were manipulated. Then ADP-ribosylation assays were conducted using these mutants to monitor FITC-labeled actin (Fig 5B). We also assessed whether there were any differences in ADP-ribosylation between α- and β/γ-actin. As shown in Fig 5B, mutants of CPILE-a showed some differences in their ADP-ribosylation of α- and β/γ-actin.

### ART assay against α-actin

K353A, E380A, and E382A mutants showed drastic reductions in ART activity (96, 92 and 99% loss, respectively). On the other hand, Y252A, E266G/N267G (PT-I), and Y377A showed substantial loss of ART activity (82, 70 and 75% loss, respectively). L308A and Y313A showed moderate effects (45% loss). In Ia, mutation of Tyr60 and Tyr62 in loop I reduced its ART activity, indicating this region is important for actin binding [31]. In CPILE-a, however, identical positional mutations, Leu61 and Asn63, had no effect on α-actin binding. Furthermore, neither S404A nor L405A had any effect.

As a reference, the ART activities of Ia and Ia mutants against α-actin were also examined. As reported already, loop I mutations (Y60S and Y62S), Y251A, and E-X-E motif mutations (E378A and E380S) caused loss of ART activity (83, 85, 98, 100 and 100% loss, respectively). Moreover, newly introduced mutations L306S, Y311S, and K351S also lowered ART activity (53, 30 and 28% loss, respectively). Interestingly, as φ mutants, both Y375A (Ia) and Y377A (CPILE-a) lost their ART activity (95 and 75% loss, respectively).

### ART assay against β/γ-actin

These are the first detailed analyses to compare ART activity against α-actin and β/γ-actin. Ia mutants showed 58% sensitivity to β/γ-actin compared with α-actin. However, CPILE-a mutants showed different ART activity profiles from Ia. In particular, Y252A and E266G/N267G (PT-I) had large effects on α-actin (83% and 70% loss, respectively) but almost no effects against β/γ-actin, while L308A and Y313A produced a moderate effect against α-actin (44% and 45% loss, respectively) but almost none against β/γ-actin. CPILE-a was found to ADP-ribosylate both α- and β/γ-actin, but its sensitivity to β/γ-actin was 36% compared with α-actin (Fig 5C), meaning that β/γ-actin is not as good of a substrate for CPILE-a as α-actin is.

### Two convex−concave interactions in the CPILE-a-α-actin complex model

**Fig 6:**
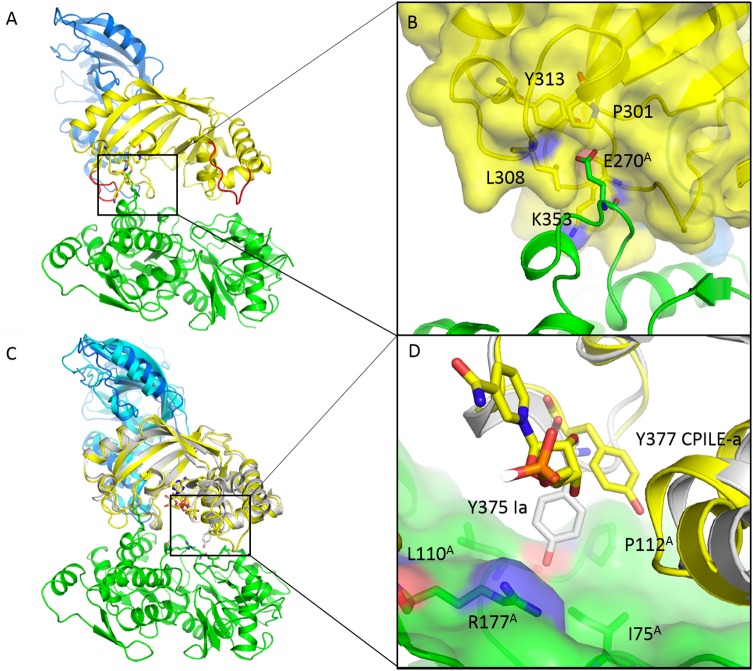
Complex structures of actin focus on convex and concave area. **A,** Model structure of the CPILE-a–α-actin complex (blue, N-terminal domain; yellow, C-terminal domain; green, actin). **B,** E270^A^ on the convex surface and the deep concave area around it. **C,** Superimposed structures of CPILE-a and Ia (cyan, N-terminal domain; gray, C-terminal domain) complexed with α-actin. The NAD–Ia–actin structure used was the post-reaction state structure from 4H03. **D,** Y375 of the Ia convex surface and the shallow concave area around it. Y377 of CPILE-a (yellow) shall change upon binding actin as does the corresponding identical residue on Ia, Y375 (gray; also see text).

Though we did extensive efforts to get CPILE-a-actin complex crystals, unfortunately we could not get any complex crystals. To verify the mutagenesis results, a modelling of the CPILE-a-α-actin complex was executed based on the crystal structure of the Ia-α-actin complex (Fig 6A and 6C). In particular, we focused on two major convex-concave interactions. One interaction used Glu270^A^ of actin as the convex surface (Fig 6A and 6B), while the other used φ convex on the ARTT-loop of CPILE-a (Fig 6C and 6D). In the former interaction, the concave surface of CPILE-a was made of Pro301, Leu308, Tyr313, and Lys353 (aliphatic region of the Lys side chain). Glu270^A^ was located on the top of the convex surface and interacted with Tyr313 on the concave surface (Fig 6B), and the hydrophobic part of the Glu270^A^ side chain was recognized by Leu308 (Fig 6B). Other surrounding interactions assist formation of the complex. I348-K355 of loop IV (including Lys353) interacts with residues of subdomain III and IV, including Met176^A^, Ala272^A^, and Glu276^A^ (the former two residues are different in β/γ-actin, as mentioned in the discussion).

The other convex−concave interaction uses φ convex on the ARTT-loop of CPILE-a (Fig 6C and 6D). The concave surface was made of His73^A^, Ile75^A^, Leu110^A^, Pro112^A^ and Arg177^A^. In the Ia-α-actin structure (pre-reaction state), Tyr375 binds the concave surface but leaves off without actin in the post-reaction state [5, 31, 32]. If this is true for CPILE-a, then Tyr377 of CPILE-a shall bind the concave surface of α-actin. However, this concave surface is a rather broad space for Tyr377; thus, the interaction does not seem as strong.

## Discussion

Interestingly, CPILE-a showed different sensitivities towards α- and β/γ-actin. Against α-actin, the maximum activity of CPILE-a was the same as that of Ia. However, the maximum activity of CPILE-a towards β/γ-actin was 36% compared with α-actin. Thus, discussion of the role of mutated residues was only based on α-actin results in the next section. In contrast, Ia showed somewhat different sensitivity compared with CPILE-a and the sensitivity towards β/γ-actin is 58% that for α-actin.

### Mutagenesis results based on CPILE-a−α-actin

PT-I and PT-II are unique CPILE-a-specific regions. From the results, it may concluded that PT-I is essential for α-actin binding. The structure of PT-I looks like a large hook. Conversely, the PT-II mutant does not show a large effect. Loop I is an important region for α-actin binding in the N-terminal domain as shown by Ia mutants [32]. Leu61 and Asn63 of CPILE-a showed almost no effect on α- or β/γ-actin binding. These residues are not conserved in CPILE-a and Ia. Furthermore, they form an α-helix in Ia. These differences may affect actin binding.

Though the function of Tyr251 (Ia) in loop II is not fully understood, this residue is flexible and important for ART activity. In CPILE-a, Tyr252 was an important residue with unknown function. Leu308 and Tyr313 mutations in loop III moderately affected ART activity (about 50% loss), and both reside in the concave surface which fit Glu-270^A^. The K353A (CPILE-a) mutation in loop IV largely affected ART activity (96% loss), and also contributed to the Glu270^A^ convex−concave interaction. In contrast, mutation of the identical residue on Ia (K351S) had a moderate effect on its activity (23% loss). Three loop V mutants (Y377A, E380A, and E382A on ARTT-loop) of CPILE-a had large effects on ART activity (75, 92, 99% loss, respectively), as corresponding identical residue mutations in Ia did. It has been suggested that Glu382 is essential for nicotinamide cleavage, while Glu380 is supposed to be important for target recognition Arg177^A^ [45]. Furthermore, these residues are very important in Ia, as well.

### Glu270^A^ convex−concave interaction

Glu270^A^ in the convex surface is important for the convex−concave interaction between CPILE-a and actin. This residue is in the so-called hydrophobic plug, which connects three actin molecules together in an actin filament [46, 47]. Glu270^A^ is also the hinge region between actin subdomains III and IV. Recently, this residue was confirmed to be crucial for ADP-ribosylation [48]. L308A, Y313A, and K353A are located on the corresponding concave surface. The roles of these residues were addressed for the first time by the present study. The K353A mutant lost its ART activity against both α- and β/γ-actin. On the other hand, L308A and Y313A mutations moderately reduced ART activity against α-actin. These identical mutations in Ia (L306S, Y311S, and K351S) also showed a similar results but in moderately effect.

### φ Convex-concave interaction

Han *et al*. proposed the ARTT motif is crucial to recognizing the substrate amino acid in the C3 exoezyme and actin-specific ARTs [49]. The consensus sequence for the ARTT motif is φ-X-X-(E/Q)-X-E, where φ represents an aromatic residue [49]. It was proposed that the φ on the first turn is important for substrate recognition, while the E/Q on the second loop is crucial for recognition of the target residue [49]. In actin-specific ARTs, the first Glu on the second loop is thought to be essential for recognition of Arg177 of the modified actin. On the other hand, Gln is thought to be essential for recognition of Asn41 in RhoA-specific ARTs.

However, the interactions with φ seem weak in crystal structures of the Ia-actin complex [31]. Later in the pre- and post-reaction state, both complex structures were determined [32]. In the pre-reaction state (NAD-bound Ia-actin), but not in the post-reaction state Tyr375 is bound to actin. Conversely, in the recently determined crystal structure of C3-RhoA, the ARTT-motif of C3 was shown to recognize the target protein RhoA and the specific residue, Asn41 for the first time [33]. Tyr180 (φ) sticks into the convex RhoA hydrophobic cavity and Gln183 (C3) interacts with Asn41. Therefore, we tried to confirm the role of the φ interaction in type IV ARTs. These residues are conserved among CPILE-a, Ia, and CDTa. They harbor Tyr in this position while C2-I, Isp2b, and SpvB/C hold Phe. Moreover, the φ mutants of CPILE-a and Ia lost their activity against both α- and β/γ-actin (CPILE-a: 75 and 38% loss, respectively/ Ia: 94 and 76% loss, respectively). This is the first report to confirm that the φ is a very important residue for substrate binding of actin-, as well as RhoA-specific ARTs.

### β/γ-Actin recognition by CPILE-a and Ia

Ia ADP-ribosylates both α- and β/γ-actin. However, C2-I only ADP-ribosylates β/γ-actin. This variation in specificity has not been explained well [50]. CPILE-a ADP-ribosylates both α- and β/γ-actins, similar to Ia, but its sensitivity towards β/γ-actin is low. The crystal structures of α-actin (4H03: NAD^+^-Ia-α-actin) and β/γ-actin (2BTF: profilin-β-actin) shows no significant structural difference (RMSD 0.52 Å) [32, 51]. Among the 18 residues difference (except the N-terminal differences) only Leu176^bA^ (Met in α-actin), Cys272^bA^ (Ala in α-actin), and Phe279^bA^ (Tyr in α-actin) are located on the actin binding surface. In particular, Cys272^bA^ is part of the Glu270^A^ convex surface. Furthermore, Ala260^bA^ and Leu267^bA^ are also part of the convex surface but they are located inside the protein. The sensitivity differences of CPILE-a towards α- and β/γ-actin may be explained by the environmental differences of Glu270^A^ on the convex surface within each actin.

In previous studies, site-directed mutagenesis was restricted to the NAD binding region of the iota toxin molecule [5, 27, 52]. After publication of Ia-actin structures, site-directed mutagenesis was also applied to the actin binding surface [31, 32]. However, which amino acid residues are crucial for the toxin-catalyzed ADP-ribosylation reaction have not been analyzed yet. Recently, Belyy *et al*. showed that Asp179^A^, which has been proposed to be crucial for ADP-ribosylation [53], did not affect Ia ART activity[48]. Conversely, Glu270^A^ was shown to be a crucial residue for ADP-ribosylation and docking site of Ia [48]. They also mentioned that the KRK sequence (Lys351, Arg352, and Lys353) in Ia is probably an important interaction site. This KRK is conserved in CPILE-a. In the present study, we confirmed that Glu270^A^ interacted with Leu308, Tyr313, and Lys353 on the concave surface. Lys353 of CPILE-a is the corresponding identical residue of Lys351 of Ia in the KRK sequence. Altogether, we showed that Glu-270^A^ on the convex surface and the concave regions of both CPILE-a and Ia are crucial to their ADP-ribosylation.

CPILE is a novel binary enterotoxin. Herein, we revealed the crystal structures of apo-CPILE-a, NAD^+^-CPILE-a and NADH-CPILE-a and compared to Ia. The structure of NAD^+^ binding cleft and the binding residues were conserved among them. CPILE-a has ART activity against α- and β/γ-actin, but shows 36% sensitivity towards β/γ-actin compared with α-actin. Furthermore, CPILE-a has low NADase activity relative to that of Ia which is an another major difference between them. There are several conserved residues around NAD^+^ binding site thus it is still open question what factors affect NADase activity.

We also characterized several mutations on the α- and β/γ-actin-binding surface and concluded that PT-I and two convex-concave interactions are essential for their modification by CPILE-a. These characterizations showed that CPILE-a is a unique ART. Another research group reported apo-BECa and NADH-BECa while we are preparing our manuscript [44]. However, the BEC-a studies did not emphasize the unique structural difference and function of PT-I, PT-II, and loop I. According to the interesting differences of the three CPILE-a and two BECa structures, only NADH-BEC-a showed a large conformational change of an ARTT loop including Tyr377. The Tyr (Tyr377 in CPILE-a and Tyr375 in Ia) on the loop position of NADH-BEC-a was also different from that of NAD^+^-Ia-actin complex and thus it is unlikely that it reflects the natural structure. However, it reflects the flexibility of the ARTT-loop. It should be noted that the closest water molecule (W1) of the C1’-N glycosyl bond in NADH-BECa structure was not observed in NAD^+^-CPILE-a structure.

Our study steps forward by crystallography and structure-based mutagenesis. We not only proved that each ART-actin has some variations in protein-substrate recognition but also suggested that the two convex-concave interactions are common and important in all family toxins.

## Abbreviations

ART: ADP-ribosyltransferase
ARTT-loop: ADP-ribosylating toxin turn turn loop
Ia/Ib: iota toxins-a/b
CPE: *Clostridium perfringens* enterotoxin
CPILE-a/b: *C*. *perfringens* iota-like enterotoxin-a/b
PT-I/II: protruding loops-I/II
C2/C3: *C*. *botulinum* toxins 2/3
CDT: *C*. *difficile* toxin
φ: an aromatic residue on the first turn of the ARTT-loop
Isp2b: *B*. *laterosporus* ADP-ribosylating toxin
a superscripted “A”: refers to an α-actin residue
a superscript “bA”: refers to a β/γ-actin residue
